# A Comparative Evaluation of Low-Level Laser and Topical Steroid Therapies for the Treatment of Erosive-Atrophic Lichen Planus

**DOI:** 10.3889/oamjms.2015.072

**Published:** 2015-08-07

**Authors:** Hanaa M. El Shenawy, Amany Mohy Eldin

**Affiliations:** *Orodental Division Department, National Research Centre, Cairo, Egypt*

**Keywords:** low level laser therapy (LLLT), steroids, oral lichen planus (OLP), visual analogue scale

## Abstract

**BACKGROUND:**

Oral lichen planus (OLP) is a chronic inflammatory disease that causes bilateral white striations, papules, or plaques on the buccal mucosa, tongue, and gingivae. Erythema, erosions, and blisters may or may not be present. Several empirical therapies have been used in the treatment of (OLP).

**OBJECTIVE:**

To evaluate the effect of low level laser therapy (LLLT) versus topical steroids for the treatment of erosive-atrophic lichen planus.

**SUBJECTS AND METHODS:**

Twenty-four patients with erosive-atrophic (OLP) were categorized into two groups. In the first group patients were treated with 970 nm diode laser irradiation, while, in the second group patients used topical corticosteroids (0.1% triamcinolone acetonide orabase). The gender, medical history and pain score were recorded. The pain score was measured before and after treatment by visual analogue scale (VAS).

**RESULTS:**

Steroid-treated group (0.1% triamcinolone acetonide orabase) show reduced pain score than laser group.

**CONCLUSION:**

Topical steroids are more effective than LLLT. LLLT may be used as an alternative treatment for symptomatic OLP when steroids are contraindicated.

## Introduction

Oral lichen planus (OLP) is relatively common chronic dermatologic disease often affecting oral mucosa [1]. OLP is reported (0.5-2.2%) of population with peak incidence in (30-60 years) with female predominance of (2:1) [1]. OLP involves a cell-mediated immunologically induced degeneration of basal cell layer of epithelium [1]. The basic two types of lesion occur: totally white (keratotic) and white (keratotic) with red (atrophic, erosive, bullous) [2]. Keratotic lesions are asmyptomatic with no need of therapy, while red lesions patient feel soreness and need treatment, as well as their liability for malignant transformation [3].

Squamous cell carcinoma in most cases ranges from (0.4-2%) [4]. OLP are usually seen on the buccal mucosa, less common on the tongue, inner aspect of the lips and gingival [5]. Numerous treatment options of (OLP) include topical and systemic agents [6]. As OLP is immunologically mediated condition, corticosteroids are recommended. Topical corticosteroids abide the mainstay of therapy, and are widely accepted as the primary treatment of choice [7].

Topical corticosteroids are the main treatment with outcomes in remission and pain/soreness relief [8]. The greatest disadvantage of topical therapy for symptomatic OLP lesions, include mucosal atrophy, candidiasis, adrenal suppression, hypertension, gastrointestinal upset and hyperglycemia [9]. Considering the resistance to topical steroids in some patients and its disadvantages, other alternative effective treatment with minimal side effects seem to be vital [10]. Recently, low-level laser therapy (LLLT) has been used for treating erosive OLP with minimal side effects [11].

The Aim of this study was to compare the effect of low-level laser therapy (LLLT) (970 nm diode laser) versus topical corticosteroids in the treatment of symptomatic OLP.

## Patients and Methods

The study included twenty-four patients (18 females,6 males) with age range from (35-70 years) recruited from outpatient clinic of Oral Medicine and Periodontology Department, Faculty of Oral and Dental Medicine, Cairo University. Patients included in the study with proven diagnosis of OLP based on the basis of WHO (World Health Organization) criteria (Kramer et al.,1978) [13]. Patients excluded from the study were smokers, pregnant and lactating ladies, patients under topical and systemic steroids during the last two months and uncontrolled diabetic patients or hypertension or with positive HCV Ab were also excluded. Medical data were collected from the patients according to questionnaire of Modified Cornel Medical Index (Brightman, 2003) [14]. The study protocol was approved by Ethical Committee of the Faculty of Oral and Dental Medicine, Cairo University. An informed consent was obtained from each patient before treatment.

All participants in both groups undergo oral hygiene instructions with complete removal of plaque and calculus as they implement intraoral inflammation and intensify both extension and symptoms of OLP lesions. Patients were advised to avoid accidental trauma on soft tissues using soft bristles toothbrush. Acidic, spicy, hard, hot food and beverages are avoided. The patients were categorized into two groups: first group: 12 patients were subjected to laser sessions twice weekly with 970 nm diode laser (Sirona Dental Laser System GmbH 2005 -2008 (Fabrikstraβe 31 64625 Bensheim Germany). The second group: 12 patients treated with topical corticosteroids (0.1% triamcinolone acetonide orabase). All cases in both groups are assessed using visual analogue scale (VAS) to graduate the severity of patients’ pain ranging from 0 (no pain) to 10 (extreme pain) (Cafaro, et al., 2014) [12] before, during and after both treatments. In the laser irradiation group the exposure time was 8.0 minutes in four successive applications for two minutes each, the exposure power setting was (3.0 watt), frequency (30 hertz). The patients were treated with diode laser in a continuous non-contact mode with (320 µm) diameter fiber optic as delivery system that was directed at the affected areas with defocused mode and overlapping exposure until blanching of the treated area had occurred. The laser therapy was in the form of two sessions weekly for two months with maximum of ten sessions. After each laser session patients were advised for cold application to prevent edema. Oralcure® gel was used postoperatively. Patients in the steroid group were treated by topical corticosteroid (0.1% topical triamcinolone acetonide preparation). This treatment was repeated four times per day for four weeks and patients were followed up weekly during this period.

### Statistical Analysis

Numerical data were explored for normality by checking the distribution of data, calculating the mean and median values as well as using tests of normality (Kolmogorov-Smirnov and Shapiro-Wilk tests). Data were presented as mean and standard deviation (SD) values. For parametric data; Student’s t-test was used to compare between the two groups. For non-parametric data; Mann-Whitney U test was used to compare between the two groups. Wilcoxon signed-rank test was used to compare between pre- and post-treatment values in each group. Friedman’s test was used to compare between pre-, post-treatment and after exacerbation values in each group. Wilcoxon signed-rank test was used for pair-wise comparisons when Friedman’s test is significant. Bonferroni’s correction was applied for the pair-wise comparisons.

Qualitative data were presented as frequencies (n) and percentages (%). Chi-square test was used to compare between the two groups.

The significance level was set at P ≤ 0.05. Statistical analysis was performed with IBM (IBM Corporation, NY, USA), SPSS (SPSS, Inc., an IBM Company) Statistics Version 20 for Windows.

## Results

No statistically significant difference was shown between mean age values and gender distributions in the two groups (Table 1).

**Table 1:** Mean age values and gender distributions in the two groups

	*Corticosteroids* *(n=12)*	*Laser* *(n=12)*	*p-value*
Age (Mean, SD)	52.2 (6.4)	53.6 (13.2)	0.766

Gender (n, %)			

Females	10 (83.3)	9 (75.0)	0.615	
Males	2 (16.7)	3 (25.0)

* Significant at p ≤ 0.05

### Medical history

Seventeen patients were medically free, 2 were hypertensive, 1 diabetic, and 4 were diabetic and hypertensive. There was no statistically significant difference between mean medical histories in the two groups (Table 2).

**Table 2:** Frequencies (n), percentages (%) and results of comparison between medical histories in the two groups

	*Corticosteroids (n=12)*		*Laser (n=12)*		*p-value*

	*n*	*%*	*n*	*%*	
Hypertension	0	0.0	2	16.7	0.255
Diabetes	0	0.0	1	8.3	
Hypertension and Diabetes	3	25.0	1	8.3	
Free	9	75.0	8	66.7	

* Significant at p ≤ 0.05

### Pain (VAS scores)

Comparisons between the two groups (pre- and post-treatment) have shown that in pre-treatment, there was no statistically significant difference between the two groups. In the post-treatment, Corticosteroids group showed statistically significantly lower mean pain scores than Laser group (*p* = 0.02). At follow up, there was no statistically significant difference between the two groups (Table 3).

**Table 3:** Mean, standard deviation (SD) values and results of comparison between pain scores in the two groups

*Time*	*Corticosteroids* *(n=12)*		*Laser* *(n=12)*		*p-value*

	*Mean*	*SD*	*Mean*	*SD*	
Pre-treatment	6.8	0.9	7.0	1.8	0.807
Post-treatment	0.9	1.0	3.9	3.0	0.020*
Follow up	0.8	1.0	1.5	0.7	0.333

* Significant at P ≤ 0.05

### Comparison between the Two Groups (after exacerbation)

After exacerbation, there was no statistically significant difference between the two groups.

**Table 4:** Mean, standard deviation (SD) values and results of comparison between pain scores in the two groups after exacerbation

*Time*	*Corticosteroids* *(n=3)*		*Laser* *(n=2)*		*p-value*

	*Mean*	*SD*	*Mean*	*SD*	
After exacerbation	4.3	2.5	3.5	0.7	0.767

* Significant at p ≤ 0.05

In Corticosteroids group as well as laser group, there was a statistically significant decrease in pain scores post-treatment (*p* = 0.024, *p* = 0.043 respectively). There was no statistically significant difference between post-treatment and follow up periods; however both showed lower mean pain scores than pre-treatment scores.

**Table 5:** Mean, standard deviation (SD) values and results of comparison between pain scores pre- and post-treatment in each group

*Time*	*Corticosteroids* *(n=12)*		*Laser* *(n=12)*	

	*Mean*	*SD*	*Mean*	*SD*
Post-treatment	0.9^b^	1.0	3.9^b^	3.0
Follow up	0.8^b^	1.0	1.5^b^	0.7

*p-value*	0.024*		0.043*	

* Significant at p ≤ 0.05, Different superscripts in the same column are statistically significantly different.

**Figure 1:**
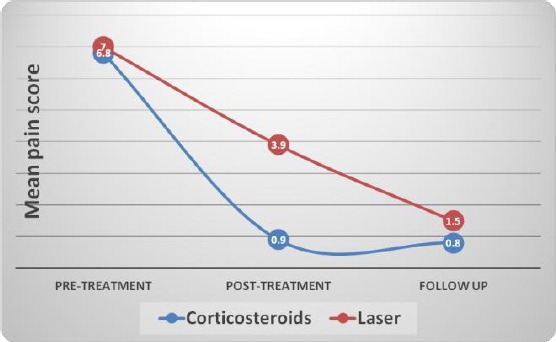
*Line chart representing changes in mean pain (VAS) scores in each group*.

### Changes after Treatment in Cases with Exacerbation

In Corticosteroids group as well as Laser group, there was a statistically significant decrease in pain scores post-treatment. There was a statistically significant increase in pain scores after exacerbation; however, there was no statistically significant difference between pain scores after exacerbation and pain scores pre-treatment.

**Figure 2:**
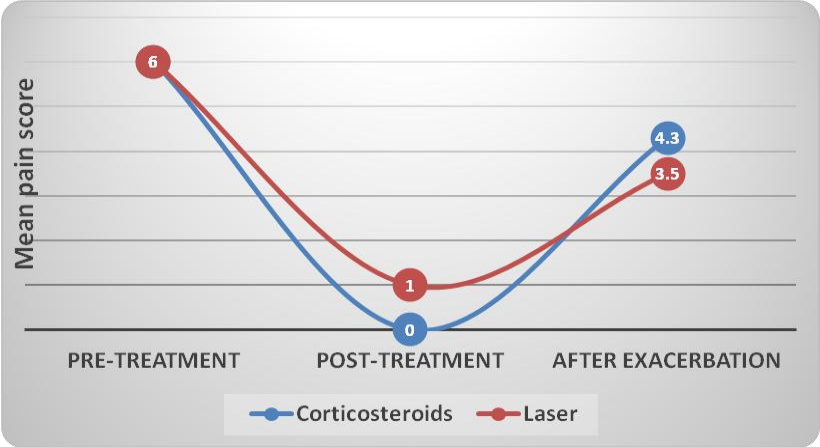
*Line chart representing changes in mean pain (VAS) scores. Pre-, post-treatment and after exacerbation in each group*.

## Discussion

OLP is a common chronic dermatologic immunological disease; its treatment remains a challenge for clinicians. Corticosteroids are the most widely accepted treatment for OLP, relieving symptoms rather than curing the disease [15]. Various treatment regimens are attempted to improve OLP lesions, but complete cure has not yet accomplished because of its recalcitrant nature [16]. Țăranu et al., 2013 [17], stated that OLP frequently occur in patients with systemic diseases such as diabetes and hypertension. The controlled diabetic patients in the study as well as controlled hypertension, undergo periodic measurements of blood pressure twice weekly, as well as random blood sugar analysis every 2 weeks were performed to monitor the systemic disease. No statistical significant difference in the medical condition between two groups (p < 0.253). Topical steroids are the most effective treatment with promising outcomes in pain/soreness relief [18], and are the main treatment for vesiculo-erosive diseases of oral mucosa including OLP to reduce pain and inflammation [19]. Triamcinolone acetonide used in the study showed a satisfactory shelf life and was accepted by OLP patients with no complications [20]. Topical steroids have common side effects include mucosal atrophy, candidiasis, adrenal suppression, gastrointestinal upset, hypertension and hyperglycemia [21]. Topical steroids have big issue in the mouth is making them adherent to oral mucosa for sufficient absorption time [22]. LLLT is a new evolution in medical/dental treatments, specifically mucocutaneous lesions such as symptomatic OLP [23]. LLLT accelerate wound healing, anti-inflammatory effects, increase cellular metabolism, modulation of immune system, vasodilatation and analgesic effects [24]. The wavelength (970 nm) selected to allow superficial action from an optical point of view. Exposure time used was 8.0 minutes. The laser group of patients assisted 2 sessions weekly as recommended by (Jajarm, 2011) [21]. Clinical condition is improved in the laser group as regard pain (VAS) was apparent after treatment showed statistical significant decrease in symptoms (p = 0.043) was detected. The results agree with statistical significant improvement of (Cafaro, 2014) (p = 0.003) [12]. The study show statistical significant decrease in pain scores in both treatment categories post-treatment. However, topical steroids show lower statistical significant mean pain scores than laser group (p = 0.02). LLLT possess physiological effects include aggregation of prostaglandins (such as PE2), immunoglobulins and lymphocytes, as well as beta-endorphin and encephalin in tissues, resulting in reduction of inflammation, immune response and pain [25].

LLLT recent treatment of OLP (Passeron T, et al., 2004) [11], (Trehan M, 2004) [26] and (Taylor CR, 2004) [26], (Köllner K, et al., 2003) [27] (Mahdavi O et al., 2013) [28]. Laser biostimulation obtain intracellular biological reactions to stimulate regenerative abilities with no side effects (Cafaro, 2014) [12]. No significant decrease after exacerbation was detected between the two groups. LLLT may be considered as an alternative treatment for OLP patients resistant to steroids, or those were corticosteroids are contraindicated. Although soft laser may not cause total relief of symptoms and disappearance of signs, but it still improves the patient’s clinical signs and symptoms providing positive influence dietary habits and quality of life.

In conclusion, the study demonstrated that topical steroid was more effective than LLLT without any adverse effects, however LLLT can be considered as an alternative treatment for symptomatic OLP in coming time and in cases where topical steroids are contraindicated.
